# Social Assistive Robots in Healthcare: Ethical Boundaries of Caritative Caring Perspective

**DOI:** 10.1111/nup.70108

**Published:** 2026-08-02

**Authors:** Malin Andtfolk, Linda Estman

**Affiliations:** ^1^ Faculty of Science and Engineering, Department of Health Sciences Åbo Akademi University Vaasa Finland; ^2^ Faculty of Health Sciences University of Stavanger Stavanger Norway

**Keywords:** caring, caritative caring theory, healthcare, social assistive robots

## Abstract

Caring is commonly described as the ethical core of nursing and as a fundamentally human way of relating to others. As social assistive robots are increasingly introduced into healthcare, questions emerge regarding the role these technologies can play in caring practices. Drawing on Katie Eriksson's theory of caritative caring, this paper examines how social assistive robots may be understood in caring and discusses the ethical boundaries that should guide their use. Caritative caring, grounded in *Caritas*, emphasizes human dignity, the care relationship, invitation, responsibility, virtue, obligation or duty, and ethical freedom. It presupposes an ethical orientation and relational openness that robots cannot possess as ethical subjects. Social assistive robots may simulate relational behaviors and support certain aspects of care, but they lack the moral agency and compassionate intentionality required for caritative caring in its full sense. At the same time, this does not mean that robots have no place in healthcare. When designed and used within a value‐sensitive and care‐oriented framework, social assistive robots may complement caregivers by supporting safety, autonomy and routine care tasks. Caritative caring theory thus offers a framework for clarifying the ethical boundaries of robot use in healthcare, not as substitutes for human compassion and responsibility, but where social assistive robots may support the conditions for human caring without replacing the relation and ethical responsibilities of caregivers.

## 1 Introduction

Caring has traditionally been understood as more than the performance of clinical tasks; within caring science it is described as an ethical and relational way of responding to human suffering (Eriksson 2006; Lindström et al. 2018). At the same time, healthcare systems are increasingly shaped by demographic change, workforce shortages and efficiency demands (World Health Organization 2000; Ministry of Economic Affairs and Employment 2022; Telang 2023). These conditions have contributed to a growing interest in technological assistance, including the use of social assistive robots (Azeta et al. 2018; Breazeal et al. 2016).

Social assistive robots are designed to interact with people through verbal and nonverbal communications and to interact socially with humans through verbal and nonverbal communication (Breazeal et al. 2016; Yousif 2020). In healthcare, these robots have been proposed as tools capable of delivering information, assisting with routine tasks, and supporting care services when needed (Tanioka et al. 2019; Kalb 2020). Research further indicates that people may experience social assistive robots as social rather than mere technical objects, especially when the robots show behaviors associated with human interaction (Ishiguro et al. 2016; Cavallo et al. 2018). This is ethically significant because perceptions of social presence may influence how caring interactions are interpreted and experienced.

At the same time, the growing use of these robots raises ethical concerns because caring is commonly understood as a deeply human phenomenon grounded in relationality, responsibility, and recognition of the other human being (Eriksson 1995; Eriksson 2006). The resulting tension concerns not only which care‐related tasks robots can perform, but whether technologically mediated interaction can meaningfully participate in caring understood as a relational and ethical practice. Discussions on robots in healthcare consequently extend beyond questions of usability and acceptance to concerns of ethically appropriate care (Beck 2016; Papadopoulos et al. 2020). Concerns related to privacy, security, and data protection have also been highlighted (Hartzog 2014; Papadopoulos et al. 2020), as well as underexplored issues such as spirituality and religious values (Hashim and Yussof 2017).

Existing discussions of robot‐mediated care often rely on an unclear understanding of caring itself (Van Aerschot and Parviainen 2020). In much of this literature, it remains unclear whether caring is understood primarily as emotional engagement, relational presence, moral responsibility, or the practical execution of care tasks. Some scholars argue that the concept of caring should be reformulated to establish a shared terminology for robot ethics (Coeckelbergh 2010; van Wynsberghe 2013), whereas others maintain that caring is an act that only humans can perform (Eriksson 2006). What remains less explored is how healthcare robotics relates to caring understood within a caritative framework grounded in Caritas, compassion, and affirmation of human dignity.

This paper offers a philosophical analysis of social assistive robots from the perspective of Eriksson's caritative caring theory. Rather than understanding caring as a set of tasks or as emotional performance, Eriksson describes caring as an ethical way of being with another person, oriented toward alleviating suffering through compassion and affirmation (Eriksson 2006, 1995; Lindström et al. 2018). Within this perspective, caring is not reducible to observable behavior alone but presupposes ethical intentionality and responsibility toward the vulnerable other. This raises a fundamental tension between the instrumental functions of robots and the ethical responsibility presupposed in caritative caring, including responsibility toward the suffering other. Against this background, the paper asks: (1) whether social assistive robots can be understood within a caritative perspective of caring, and (2) if so, how their role in healthcare should be ethically defined.

We argue that although SARs may support certain practical and relational dimensions of care, they cannot fulfil the ethical conditions of caritative caring in the full sense attributed to human caregivers. The analysis first clarifies caring and caritative caring within Eriksson's framework, before examining social assistive robots in relation to central ethical categories of caritative caring. Finally, implications for ethically oriented design and use of healthcare robotics are discussed.

## 2 Theoretical Background: Caring as a Caritative Phenomenon

To examine whether social assistive robots can meaningfully participate in caring, a clear conceptual framework is required. The present analysis draws on caring science and, more specifically, on Eriksson's theory of caritative caring (Eriksson 2006, 1995; Lindström et al. 2018). A central idea is that caring takes place in relation to another person (Eriksson 1987a, 1987b; Lindström et al. 2018). Caring involves meeting the patient as a human being rather than only responding to illness or practical needs. For Eriksson, the relationship between caregiver and patient is therefore an important part of caring itself. This relational understanding implies that caring cannot be reduced to tasks or technical competence alone. Caring also involves sensitivity to the vulnerable person.

### 2.1 Caritas and the Purpose of Caring

Caritas is central to Eriksson's theory of caritative caring and refers to love, compassion, and a willingness to respond to the suffering of another person (Eriksson 2006; Lindström et al. 2018). Within this framework, caring is understood not merely as the performance of care tasks, but as an ethical and relational response to the vulnerable human being. Caritas therefore represents an ethical orientation grounded in responsibility for the other and affirmation of human dignity (Fagerström et al. 2021; Näsman 2020).

From a caritative perspective, the patient is not primarily understood as a recipient of treatment, but as a person whose suffering and vulnerability call for compassion and ethical responsiveness. Although practical assistance and professional competence remain important, they do not alone constitute caring in a caritative sense. Caring becomes ethically meaningful through the caregiver's willingness to encounter the other person with openness, compassion, and respect for dignity. These ethical foundations become particularly important when considering whether technologically mediated forms of care can meaningfully participate in caring practices.

### 2.2 The Ethical Categories of Caritative Caring

In *Towards a Caritative Care Ethics*, Eriksson (1995) articulates seven ethical categories that structure caritative caring: *human dignity, the care relationship, invitation, responsibility, virtue, obligation or duty, and ethical freedom*. These categories are inspired in part by Nygren (1926) reflections on ethical disposition. *Human dignity* refers to the inherent and inviolable worth of every person. Within caritative caring, human dignity functions as the ethical ground from which caring responsibilities and relationships emerge. *The care relationship* and *invitation* emphasize that caring presupposes openness to the other. Ethical care involves inviting the other into a relationship grounded in respect (Eriksson 1987a; Lindström et al. 2018). *Responsibility, virtue*, and *obligation or duty* describe different aspects of caregiver's ethical stance. *Responsibility* can be understood both as an inner commitment and as a professional *obligation or duty* (Eriksson 1995). *Virtue* refers to the inner qualities that support caring action, while obligation reflects professional and ethical commitments. Caritative caring also includes *ethical freedom*. Ethical caring therefore involves more than following rules or procedures; it requires sensitivity, responsibility, and the freedom to act in ways directed toward the good of the other person. Eriksson highlights the caregiver's freedom to choose actions that promote the good of the patient (Eriksson 1995; Bergbom and Nåden 2021). These ethical conditions become particularly significant when examining whether SARs can participate in caring understood as an ethical and relational practice rather than merely as task performance.

## 3 Social Assistive Robots in Relation to the Ethical Categories of Caritative Caring

This section examines social assistive robots in relation to the ethical foundations of caritative caring. (Eriksson 1995, 2006; Lindström et al. 2018). The focus is not on whether robots can replicate human caring, but on whether they can support or whether they can meaningfully participate in caring practices shaped by these ethical conditions. Some argue that advanced AI systems may warrant moral consideration (Gunkel 2018; Schwitzgebel and Garza 2015), whereas others maintain they are tools designed for human purposes (Balkin 2017). From a caritative perspective, however, the question concerns whether robots can participate in caring understood as an ethical and relational practice grounded in Caritas. The analysis below examines social assistive robots in relation to the ethical categories of caritative caring (cc. Eriksson 1995; Nygren 1926).

### 3.1 Human Dignity

Human dignity constitutes the ethical core of caritative caring and refers to the intrinsic and inviolable worth of the person, independent of functional ability or health status. Caring actions must affirm and safeguard this dignity.

Social assistive robots may contribute indirectly to dignity, for example by promoting safety, autonomy, and access to information (van Wynsberghe 2013; Tanioka et al. 2019). In this respect, robots may contribute to conditions that can support patients in daily activities and care routines. However, affirming dignity in a caritative sense involves more than safeguarding autonomy. It entails recognizing the other as a suffering and vulnerable human being. While robots can follow programmed rules that respect privacy and autonomy, they do not relate to dignity. From a caritative standpoint, robots may therefore support conditions for dignified care without themselves participating in dignity‐affirming caring relationships.

### 3.2 The Care Relationship and Invitation

Caritative caring unfolds within a relational encounter characterized by openness, invitation, and responsiveness to the other (Routasalo and Isola 1996; Eriksson 1987a; Lindström et al. 2018). The relationship is not external to caring, but part of it.

Social assistive robots can interact socially and may be perceived as companions, especially in situations involving loneliness or isolation. This may influence how patients experience presence, companionship, and emotional support in care situations. They may also support communication and reduce loneliness in care environments (Turkle 1984; Ishiguro et al. 2016; Cavallo et al. 2018). Although robots may simulate attentiveness and responsiveness, the interaction does not emerge from mutual vulnerability or relational presence. Robots can simulate responsiveness, but they do not participate in the relational depth that characterizes caritative caring. Their contribution therefore remains supportive and indirect. They may free time and resources that allow human caregivers to engage more fully in relational care (Coco et al. 2018; Nyholm et al. 2021; Parviainen et al. 2019).

### 3.3 Responsibility, Virtue, Obligation and Duty

Caritative caring presupposes responsibility and virtue as part of the caregiver's ethical stance (Eriksson 1995). Responsibility involves both commitment and duty in relation to the suffering of the other.

Robots can be designed to follow ethical guidelines and to support patient autonomy. They may also operate within structured care processes (Hägglund et al. 2024). Although robots may operate according to predefined ethical principles or governance frameworks, responsibility in a caritative sense involves more than rule‐following or procedural compliance. It presupposes the capacity to respond ethically to the suffering other and to answer for one's actions within a relational context. Although virtue‐ethical approaches have been proposed for AI governance (Smith and Vickers 2024), this capacity cannot be cultivated in robots. They execute programmed functions; they do not bear responsibility for their actions. For this reason, responsibility for care practices involving social assistive robots ultimately remains with human caregivers, designers, and institutions (Nyholm 2023).

### 3.4 Ethical Freedom

Ethical freedom refers to the caregiver's capacity to make ethically grounded judgments guided by responsibility and concern for the other. It involves reflective judgment in concrete situations (Eriksson 1995; Bergbom and Nåden 2021).

Social assistive robots can operate with programmed ethical guidelines and safety standards (Darling 2016), but this differs from ethical freedom. Although robots may simulate decision‐making processes, they do not engage in reflective moral deliberation grounded in responsibility or compassion. Their actions remain dependent on predefined parameters and externally determined goals. From a caritative standpoint, ethical freedom remains an exclusively human capacity and cannot be delegated to technological systems.

## 4 Delimiting the Role of Social Assistive Robots in Caritative Caring

The preceding analysis suggests that social assistive robots should not be conceived as agents capable of providing caritative caring in the full sense attributed to human caregivers. From a caritative perspective, robots cannot participate in Caritas as an ethical orientation grounded in compassion, responsibility, and responsiveness toward the suffering other. This does not exclude their role in care. When designed and used within a caritative framework, social assistive robots can support caring practices by assisting with routine tasks, enhancing safety, and facilitating autonomy. In doing so, they may create conditions that allow human caregivers to engage more fully in the relational and compassionate dimensions of care.

Caritative caring theory thus supports a model in which robots enhance, rather than replace, human caregiving. Rather than adapting the concept of caring to technological capabilities, the framework helps clarify the ethical boundaries of robotic involvement in care.

### 4.1 Human‐Robot Care: Implications and Future Directions

The preceding analysis suggests that social assistive robots cannot fulfil the ethical conditions of caritative caring in the full sense. Their increasing presence in healthcare raises questions about how they should be integrated into caring practices. From a caritative perspective, the relevant is not whether robots can replace human caregivers, but how technological systems can be aligned with the ethical foundations of caring. If caring is grounded in *Caritas* (Eriksson 2006, 1995), then technology integration must be evaluated in relation to compassion, responsibility, and respect for human dignity. Robots may be particularly suited to instrumental and supportive functions, such as monitoring, information provision, and assistance with routine tasks. In these areas, they can enhance safety, autonomy, and efficiency (Tanioka et al. 2019). When such functions reduce workload, they may indirectly support the relational aspects of care by allowing more time for human presence and interaction.

An explicit ethical dimension in the development and use of care technologies remains essential (Korhonen 2017). Approaches such as care‐centered design (van Wynsberghe 2013) illustrate how technological development can be guided by normative values rather than solely by efficiency. Ongoing dialogue between designers, caregivers, patients, and society is therefore crucial to ensure that technological development remains aligned with the ethical foundations of care (Pekkarinen et al. 2020). At the same time, the increasing use of social assistive robots challenges understandings of care and professional roles within healthcare. As Locsin and Ito (2018) and Maibaum et al. (2021) suggest, the introduction of robotic technologies may prompt reconsideration of professional responsibilities and conceptual distinctions within caring practices. From a caritative standpoint, however, such development should not dilute the ethical meaning of caring. Rather than redefining caring in response to technological capabilities, the ethical foundations of caring should remain the point of reference.

The question is therefore not whether robots should be treated as moral agents, but how technological systems can be structured in ways that support rather than displace human capacities for compassion, responsibility, and relational presence.

## 5 Conclusion

This paper has examined how social assistive robots may be understood within a caritative perspective on caring and discussed the ethical boundaries that should guide their use in healthcare. Drawing on Katie Eriksson's caritative caring theory, caring was understood as an ethical and relational practice grounded in *Caritas*, and expressed through relational presence, responsibility, and ethical freedom. The analysis suggests that social assistive robots cannot provide caritative caring in the full sense attributed to human caregivers. Although robots may support care practices and simulate forms of social interaction, they do not participate in caring through ethical freedom, moral responsibility, or compassionate responsiveness in the way caritative caring presupposes. At the same time, this does not exclude their role in healthcare. Social assistive robots may support caring practices by assisting with routine tasks, contributing to safety, and supporting patient autonomy. Their role should therefore be understood as supportive and complementary rather than substitutive.

From a caritative caring perspective, the ethical boundaries of robot use become particularly important. Social assistive robots should not replace the relational and ethical dimensions of human caring, but they may be integrated in ways that support the conditions for such caring. Caritative caring theory thus provides a framework for evaluating how technological systems can be used without compromising the ethical foundations of caring.

## Funding

The authors have nothing to report.

## Ethics Statement

This study is a philosophical paper and does not include empirical research involving human participants or animals. Ethical approval and informed consent were therefore not required.

## Conflicts of Interest

The authors declare no conflicts of interest.

## Data Availability

Data sharing not applicable to this article as no datasets were generated or analysed during the current study. Not applicable. No new data were generated in this study.

## References

[nup70108-bib-0001] Van Aerschot, L. , and J. Parviainen . 2020. “Robots Responding to Care Needs? A Multitasking Care Robot Pursued for 25 Years, Available Products Offer Simple Entertainment and Instrumental Assistance.” Ethics and Information Technology 22, no. 3: 247–256. 10.1007/s10676-020-09536-0.

[nup70108-bib-0003] Azeta, J. , C. Bolu , A. A. Abioye , and O. Festus . 2018. “A Review on Humanoid Robotics in Healthcare.” MATEC Web of Conferences 153, no. 5: 1–5. 10.1051/matecconf/201815302004.

[nup70108-bib-0004] Balkin, J. M. 2017. “The Three Laws of Robotics in the Age of Big Data.” Ohio State Law Journal 78: 1217–1241.

[nup70108-bib-0007] Beck, S. 2016. “The Problem of Ascribing Legal Responsibility in the Case of Robotics.” AI and Society 31, no. 4: 473–481. 10.1007/s00146-015-0624-5.

[nup70108-bib-0008] Bergbom, I. , D. Nåden , and L. Nyström . 2021. “Katie Eriksson's Caring Theories. Part 1. The Caritative Caring Theory, the Multidimensional Health Theory and the Theory of Human Suffering.” Scandinavian Journal of Caring Sciences 36, no. 3: 782–790. 10.1111/scs.13036. 34609017

[nup70108-bib-0087] Breazeal, C. , K. Dautenhahn , and T. Kanda . 2016. “Social Assistive Robotics.” In Springer Handbook of Robotics. Springer, 1935–1972. 10.1007/978-3-319-32552-1_72.

[nup70108-bib-0012] Cavallo, F. , R. Esposito , R. Limosani , et al. 2018. “Robotic Services Acceptance in Smart Environments With Older Adults: User Satisfaction and Acceptability Study.” Journal of Medical Internet Research 20, no. 9: e264. 10.2196/jmir.9460. 30249588 PMC6231879

[nup70108-bib-0014] Coco, K. , M. Kangasniemi , and T. Rantanen . 2018. “Care Personnel's Attitudes and Fears Toward Care Robots in Elderly Care: A Comparison of Data From the Care Personnel in Finland and Japan.” Journal of Nursing Scholarship 50, no. 6: 634–644. 10.1111/jnu.12435. 30354007

[nup70108-bib-0015] Coeckelbergh, M. 2010. “Health Care, Capabilities, and Ai Assistive Technologies.” Ethical Theory and Moral Practice 13, no. 2: 181–190. 10.1007/s10677-009-9186-2.

[nup70108-bib-0086] Darling, K. 2016. “Extending Legal Protection to Social Robots: The Effects of Anthropomorphism, Empathy, and Violent Behavior Towards Robotic Objects.” In Robot law, edited by R. Calo , A. M. Froomkin , and I. Kerr , 213–232. Edward Elgar Publishing.

[nup70108-bib-0018] Eriksson, K. 1987a. The Break: A Depiction of the Knowledge Object in Caring Sciences. Libris.

[nup70108-bib-0019] Eriksson, K. 1987b. The Idea of Caring. Almqvist & Wiksell.

[nup70108-bib-0025] Eriksson, K. 2006. The Suffering Human Being, 2051. Nordic Studies Press.

[nup70108-bib-0026] Eriksson, K. 1995. Towards a Caritative Care Ethics. Reports From the Department of Caring Science 5/1995, edited by K. Eriksson , 9–39. Åbo: Åbo Academi University, Vårdforskning [Care research].

[nup70108-bib-0028] Fagerström, L. M. , J. Hemberg , C. Koskinen , et al. 2021. “The Core of Katie Eriksson's Caritative Caring Theory—A Qualitative Study From a Postdoctoral Perspective.” Scandinavian Journal of Caring Sciences 35, no. 4: 1240–1249. 10.1111/scs.12942. 33301618

[nup70108-bib-0033] Gross, J. 2020. “Interviewing Roomba: A Posthuman Study of Humans and Robot Vacuum Cleaners.” Explorations in Media Ecology 19, no. 3: 285–297. 10.1386/eme_00047_1.

[nup70108-bib-0035] Gunkel, D. 2018. Robot Rights. Cambridge: MIT Press.

[nup70108-bib-0036] Hägglund, S. , M. Andtfolk , S. Rosenberg , M. Wingren , S. Andersson , and L. Nyholm . 2024. “Do You Wanna Dance? Tales of Trust and Driving Trust Factors in Robot Medication Counseling in the Pharmacy Context.” Frontiers in Robotics and AI 11: 1332110. 10.3389/frobt.2024.1332110. 39170902 PMC11336249

[nup70108-bib-0037] Hartzog, W. 2014. “Unfair and Deceptive Robots.” Maryland Law Review 74, no. 4: 785–829. https://digitalcommons.law.umaryland.edu/cgi/viewcontent.cgi?article=3675&context=mlr.

[nup70108-bib-0038] Hashim, R. , and H. Yussof . 2017. “Humanizing Humanoids Towards Social Inclusiveness for Children With Autism.” Procedia Computer Science 105: 359–364. 10.1016/j.procs.2017.01.234.

[nup70108-bib-0040] Ishiguro, K. , Y. Majima , and N. Sakata . 2016. “Deployment of Arcs Model and Utilization of Communication Robot in Patient Education.” Proceedings of the 9th International Joint Conference on Biomedical Engineering Systems and Technologies 5: 371–376. 10.5220/0005774603710376.

[nup70108-bib-0041] Kalb, C. 2020. Could a Robot Care for Grandma? National Geographic. https://www.nationalgeographic.com/magazine/article/could-a-robot-care-for-grandma-feature.

[nup70108-bib-0044] Korhonen, E.‐S. 2017. “Technology and Its Ethics.” Doctoral diss., Åbo Akademi University. https://urn.fi/URN:ISBN:978-952-12-3541-2.

[nup70108-bib-0047] Lindström, U. Å. , L. L. Nyström , J. E. Zetterlund , and K. Eriksson . 2018. “Theory of Caritative Caring.” In Nursing Theorists and Their Work, edited by M. R. Alligood , (9th ed., 448–461). Mosby Elsevier.

[nup70108-bib-0048] Locsin, R. C. , and H. Ito . 2018. “Can Humanoid Nurse Robots Replace Human Nurses?” Journal of Nursing Research 5, no. 1: 1–6. 10.7243/2056-9157-5-1.

[nup70108-bib-0050] Maibaum, A. , A. Bischof , J. Hergesell , and B. Lipp . 2021. “A Critique of Robotics in Health Care.” AI and Society 37: 467–477. 10.1007/s00146-021-01206-z.

[nup70108-bib-0051] Ministry of Economic Affairs and Employment . 2022. Occupational Barometer: Increase in Labour Shortages has Slowed Down but Health and Social Services Continue to Account for Top Shortage Occupations. https://tem.fi/en/-/occupational-barometer-increase-in-labour-shortages-has-slowed-down-but-health-and-social-services-continue-to-ac-count-for-top-shortage-occupations.

[nup70108-bib-0053] Näsman, Y. 2020. “The Theory of Caritative Caring: Katie Eriksson's Theory of Caritative Caring Presented From a Human Science Point of View.” Philosophers for Nursing 21, no. 4: e12321. 10.1111/nup.12321. 32974999

[nup70108-bib-0055] Nygren, A. 1926. Etiska grundfrågor. [Basic ethical questions]. Almqvist & Wiksell.

[nup70108-bib-0056] Nyholm, L. , R. Santamäki‐Fischer , and L. Fagerström . 2021. “Users’ Ambivalent Sense of Security With Humanoid Robots in Healthcare.” Informatics for Health and Social Care 46, no. 2: 218–226. 10.1080/17538157.2021.1883027. 33627020

[nup70108-bib-0057] Nyholm, S. 2023. This is Technology Ethics: An Introduction (First Edition). JohnWiley and Sons, Inc.

[nup70108-bib-0058] Papadopoulos, I. , C. Koulouglioti , R. Lazzarino , and S. Ali . 2020. “Enablers and Barriers to the Implementation of Socially Assistive Humanoid Robots in Health and Social Care: A Systematic Review.” BMJ Open 10, no. 1: e033096. 10.1136/bmjopen-2019-033096. PMC6955545 31924639

[nup70108-bib-0059] Parviainen, J. , T. Turja , and L. Van Aerschot . 2019. “ *Social Assistive Robots and Human Touch in Care: The Perceived Usefulness of Robot Assistance Among Healthcare Professionals* .” In Social Assistive Robots: Technological, Societal and Ethical Aspects of Human–Robot Interaction, edited by O. Korn , 187–204. Springer. 10.1007/978-3-030-17107-0_10.

[nup70108-bib-0060] Pekkarinen, S. , L. Hennala , O. Tuisku , et al. 2020. “Embedding Care Robots into Society and Practice: Socio‐Technical Considerations.” Futures 122: 102593. 10.1016/j.futures.2020.102593.

[nup70108-bib-0066] Routasalo, P. , and A. Isola . 1996. “The Right to Touch and Be Touched.” Nursing Ethics 3, no. 2: 165–176. 10.1177/096973309600300209. 8717880

[nup70108-bib-0067] Schwitzgebel, E. , and M. Garza . 2015. “A Defense of the Rights of Artificial Intelligences: Defense of the Rights of Artificial Intelligences.” Midwest Studies In Philosophy 39: 98–119. 10.1111/misp.12032.

[nup70108-bib-0070] Smith, N. , and D. Vickers . 2024. “Living Well With AI: Virtue, Education and Artificial Intelligence.” Theory and Research in Education 22, no. 1: 19–44. 10.1177/14778785241231561.

[nup70108-bib-0072] Tanioka, T. , M. C. Smith , K. Osaka , and Y. Zhao . 2019. “Framing the Development of Humanoid Healthcare Robots in Caring Science.” International Journal for Human Caring 23, no. 2: 112–120. 10.20467/1091-5710.23.2.112.

[nup70108-bib-0073] Telang, S. 2023. Finland Faces Stiff Competition As Western Countries Strive To Attract International Nurses. Helsinki Times. https://www.helsinkitimes.fi/finland/finland-news/domestic/23241-finland-faces-stiff-competition-as-western-countries-strive-to-attract-international-nurses.html.

[nup70108-bib-0077] Turkle, S. 1984. The Second Self: Computers And The Human Spirit. Simon and Schuster.

[nup70108-bib-0080] World Health Organization . 2000. World Health Report, 2000, Health Systems: Improving Performance. https://apps.who.int/iris/bitstream/handle/10665/42281/WHR_2000-eng.pdf?sequence=1&isAllowed=y.

[nup70108-bib-0082] World Health Organization . 2024. WHO calls for urgent transformation of care and support systems for older people. https://www.who.int/news/item/01-10-2024-who-calls-for-urgent-transformation-of-care-and-support-systems-for-older-people.

[nup70108-bib-0084] van Wynsberghe, A. 2013. “Designing Robots for Care: Care Centered Value‐Sensitive Design.” Science and Engineering Ethics 19, no. 2: 407–433. 10.1007/s11948-011-9343-6. 22212357 PMC3662860

[nup70108-bib-0085] Yousif, J. 2020. “Humanoid Robot as Assistant Tutor for Autistic Children.” International Journal of Computation and Applied Sciences 8, no. 2: 8–13. https://ssrn.com/abstract=3616810.

